# Association between underweight and pulmonary function in 282,135 healthy adults: A cross-sectional study in Korean population

**DOI:** 10.1038/s41598-019-50488-3

**Published:** 2019-10-04

**Authors:** Jong Geol Do, Chul-Hyun Park, Yong-Taek Lee, Kyung Jae Yoon

**Affiliations:** 1 Department of Physical and Rehabilitation Medicine, Kangbuk Samsung Hospital, Sungkyunkwan University School of Medicine, Seoul, Republic of Korea; 2 Department of Rehabilitation Medicine, Seoul National University Hospital College of Medicine, Seoul, Republic of Korea

**Keywords:** Malnutrition, Risk factors

## Abstract

In contrast to obesity, studies on the relationship between underweight and pulmonary function are still sparse. Thus, the objective of this study was to investigate the effect of being underweight on pulmonary function in a general population without apparent lung disease. A total of 282,135 retrospective cohort subjects between January 2012 and December 2014 in Korea were included. Using multivariate-adjusted analysis, the relationship between body mass index (BMI) and pulmonary function were assessed. Underweight individuals represented 5.5% of the total study population (n = 282,135), with most (87.9%) of them being females. Compare to normal weight and obese, underweight was associated with decreased pulmonary function. Forced expiratory volume in first second (FEV1), predicted FEV1 (%), forced vital capacity (FVC), predicted FVC (%), and peak expiratory flow (PEF) were lower in the underweight group than those in other groups after adjusting for age, sex, height, status of smoking, frequency of vigorous exercise, diabetes, and high-sensitivity C-reactive protein (hsCRP) (*P* < 0.001). Lower BMI tended to decrease pulmonary function parameters such as FEV1 (L), predicted FEV1 (%), FVC (L), predicted FVC (%), and PEF (L/sec) (*P* for trend <0.001). After adjusting for possible confounders, odds ratios (ORs; 95% confidence interval) for subjects with predicted FEV1% < 80% in underweight and normal weight groups compared to obese group (reference) were 2.10 (1.98–2.21), and 0.93 (0.90–0.97), respectively. ORs for subjects with predicted FVC% < 80% in underweight and normal weight groups compared to obese group (reference) were 4.90 (4.62–5.18) and 1.32 (1.27–1.38), respectively. This study demonstrated a proportional relationship between pulmonary function and the degree of BMI. We found that underweight status was independently associated with decreased pulmonary function in Korean population.

## Introduction

The association between body weight and health condition is one of the most important issues in public health. It has received considerable attention. Many studies have focused on effects of obesity on pulmonary function^1–3^. In contrast, relatively few studies have assessed the association between underweight and pulmonary function. Previous studies have shown that functional residual capacity and expiratory reserve volume are significantly decreased in overweight and obesity conditions^4^. It is known that obese individuals have higher risk of respiratory impairments such as breathlessness and airway dysfunction^1,5^. However, the relationship between underweight and pulmonary function is still a matter of debate.

Underweight status is associated with increased morbidity and mortality^6^. In a recent cohort study, an increased mortality risk is observed in underweight individuals^7^. In case of acute respiratory distress syndrome (ARDS) or acute lung injury (ALI), high mortality rate is also observed in underweight patients^8^. Moreover, previous reports have shown association between underweight and morbidity^9,10^. However, due to the relative lack of attention, effects of underweight status on respiratory system have been rarely studied. Several studies have explored the relationship between respiratory function and underweight^11,12^. Dynamic lung functions such as forced vital capacity (FVC) and forced expiratory volume in first second (FEV1) are poor in underweight female young adults^11^. In a study of 327 children and adolescent population, underweight participants have lower pulmonary function in predicted FVC and vital capacity (VC)^12^. However, previous studies have been conducted on subjects with young age groups, making it difficult to accurately predict the effect of underweight on pulmonary function in a general population. To date, the relationship between underweight and pulmonary function among adults has been largely unexplored. Thus, the objective of the present study was to examine the association between underweight and pulmonary function in a general population without apparent lung disease.

## Results

### Baseline characteristics of participants

Baseline clinical and biochemical characteristics of subjects are presented in Table 1. Subjects were categorized according to body mass index (BMI). There were a total of 282,135 participants, including 152,306 men and 129,829 women. Mean age of all subjects was 39.8 ± 9.3 years. The obese group had more unfavorable clinical characteristics and biochemical profiles than other groups. Frequencies of hypertension, diabetes and values of weight, height, waist circumference, glycated hemoglobin (HbA1c), glucose, fasting insulin, total cholesterol, triglyceride, low-density lipoprotein-cholesterol (LDL-C), and homeostatic model assessment of insulin resistance (HOMA-IR) were significantly higher in the obese group (*P* < 0.001). Frequency of vigorous exercise was significantly lower in the underweight group compare to that in other groups (*P* < 0.001).

**Table 1:** Clinical characteristics of a general Korean population used in this study (n = 282,135).

	BMI category			*P* value
	Underweight (n = 15,462)	Normal (n = 188,490)	Obese (n = 78,183)	
Sex				
Female (%)	87.9	52.2	22.9	<0.001
Male (%)	12.1	47.8	77.1	
Age (years)	35.4 ± 7.8	39.6 ± 9.3	41.0 ± 9.3	<0.001
BMI (kg/m^2^)	17.6 ± 0.7	22.0 ± 1.8	27.4 ± 2.2	<0.001
Glucose (mg/dl)	88.9 ± 9.2	93.7 ± 13.1	100.1 ± 18.3	<0.001
Total cholesterol (mg/dl)	179.2 ± 28.9	191.5 ± 33.0	204.5 ± 35.6	<0.001
Triglyceride (mg/dl)	67.7 ± 28.0	98.1 ± 62.2	153.0 ± 98.0	<0.001
HDL-C (mg/dl)	70.6 ± 14.4	60.4 ± 14.6	50.2 ± 12.0	<0.001
LDL-C (mg/dl)	98.5 ± 24.6	116.4 ± 30.6	131.6 ± 32.0	<0.001
Albumin (g/dl)	4.56 ± 0.2	4.55 ± 0.2	4.58 ± 0.2	<0.001
Total protein (g/dl)	7.29 ± 0.4	7.26 ± 0.4	7.31 ± 0.4	<0.001
HbA1c (%)	5.5 ± 0.3	5.6 ± 0.4	5.8 ± 0.6	<0.001
Fasting insulin (uIU/ml)	4.0 ± 2.2	5.3 ± 4.6	8.8 ± 5.5	<0.001
hsCRP (mg/L)	0.07 ± 0.42	0.09 ± 0.30	0.15 ± 0.33	<0.001
Height (cm)	163.2 ± 6.4	166.5 ± 8.4	169.9 ± 8.3	<0.001
Weight (kg)	47.0 ± 4.2	61.3 ± 8.8	79.2 ± 10.1	<0.001
Waist circumference (cm)	66.5 ± 3.9	78.4 ± 6.5	92.1 ± 6.6	<0.001
SBP (mmHg)	97.3 ± 9.5	105.1 ± 12.1	115.2 ± 12.4	<0.001
DBP (mmHg)	63.8 ± 7.9	68.2 ± 9.5	74.7 ± 10.1	<0.001
Smoking (pack-year)	1.2 ± 4.7	3.8 ± 7.7	7.2 ± 9.7	<0.001
Smoking				
Never smoked (%)	86.3	64.9	43.1	<0.001
Former smoker (%)	5.6	16.2	24.6	
Current smoker (%)	8.1	18.8	32.3	
Vigorous exercise (times/week)	0.6 ± 1.3	0.9 ± 1.5	1.0 ± 1.5	<0.001
Hypertension (%)	1.3	6.1	16.8	<0.001
Diabetes (%)	0.6	2.1	4.8	<0.001
HOMA-IR	0.9 ± 0.5	1.2 ± 1.1	2.2 ± 1.7	<0.001

Data are presented as percentage or mean ± standard deviation.

BMI categories: normal (between 18.5 and 25 kg/m^2^), underweight (<18.5 kg/m^2^), and obese (≥25 kg/m^2^).

*BMI*, body mass index; *HDL-C*, high-density lipoprotein cholesterol; *LDL-C*, low-density lipoprotein cholesterol; *HbA1c*, glycated hemoglobin; *hsCRP*, high-sensitivity C-reactive protein; *SBP*, systolic blood pressure; *DBP*, diastolic blood pressure; *HOMA-IR*, homeostasis model assessment of insulin resistance.

### Pulmonary function parameters

Table 2 compares pulmonary function parameters of study groups. Regarding spirometric values, FEV1 (L), predicted FEV1 (%), FVC (L), predicted FVC (%), and peak expiratory flow (PEF) (L/sec) were lower in the underweight group than those in other groups. In contrast, FEV1/FVC was significantly higher in the underweight group compare to other groups (*P* < 0.001). Proportions of subjects with predicted FEV1% < 80% and predicted FVC% < 80% were significantly higher in the underweight group compare to other groups (*P* < 0.001).

**Table 2:** Pulmonary function parameters of the study group according to BMI.

	BMI category			*P* value
	Underweight (n = 15,462)	Normal (n = 188,490)	Obese (n = 78,183)	
FEV1 (L)	2.84 ± 0.5	3.23 ± 0.7*	3.47 ± 0.7*	<0.001
Predicted FEV1 (%)	87.96 ± 10.15	91.82 ± 10.1*	91.19 ± 10.2*	<0.001
Predicted FEV1% < 80%	3,160 (20.4)	21,235 (11.3)*	9,932 (12.7)*	<0.001
FVC (L)	3.19 ± 0.6	3.85 ± 0.9*	4.24 ± 0.8*	<0.001
Predicted FVC (%)	84.79 ± 10.3	93.75 ± 10.7*	95.00 ± 10.3*	<0.001
Predicted FVC% < 80%	4,445 (28.7)	13,248 (7.0)*	3,568 (4.6)*	<0.001
FEV1/FVC	89.12 ± 6.6	84.23 ± 6.8*	82.06 ± 5.5*	<0.001
FEV1/FVC < 70%	136 (0.9)	4,053 (2.2)*	1,953 (2.5)*	<0.001
PEF (L/sec)	6.31 ± 1.3	7.59 ± 2.0*	8.59 ± 2.0*	<0.001

Data are presented as mean ± standard deviation or numbers of subjects (%).

BMI categories: normal (between 18.5 and 25 kg/m^2^), underweight (<18.5 kg/m^2^), and obese (≥25 kg/m^2^).

^*^P < 0.001 to underweight.

*BMI*, body mass index; *FEV1*, Forced expiratory volume in first second; *FVC*, forced vital capacity; *PEF*, peak expiratory flow.

Adjusted mean values of parameters related to pulmonary function are presented in Table 3. Data were adjusted for multiple covariates such as age, sex, height, status of smoking, vigorous exercise (times/week), diabetes, and hsCRP. After adjusting for multiple covariates, the underweight group had lower FEV1 (L), predicted FEV1 (%), FVC (L), predicted FVC (%), and PEF (L/sec) than other groups (*P* < 0.001). Groups with lower BMI tended to have decreased pulmonary function parameters such as FEV1 (L), predicted FEV1 (%), FVC (L), predicted FVC (%), and PEF (L/sec) (*P* for trend < 0.001). FEV1/FVC tended to decrease as BMI increased (*P* for trend <0.001). FEV1/FVC was higher in the underweight group than other groups (*P* < 0.001) (Fig. 2).

**Table 3:** Adjusted mean values of pulmonary function parameters (95% CI) in the study group according to BMI.

	BMI category			*P* for trend	*P* value
	Underweight (n = 15,462)	Normal (n = 188,490)	Obese (n = 78,183)		
FEV1 (L)	3.12 (3.11–3.13)	3.28 (3.28–3.29)^*^	3.28 (3.27–3.28)^*^	<0.001	<0.001
Predicted FEV1 (%)	86.49 (86.27–86.72)	90.91 (90.77–91.05)^*^	90.73 (90.58–90.88)^*^	<0.001	<0.001
FVC (L)	3.62 (3.61–3.62)	3.91 (3.90–3.91)^*^	3.96 (3.95–3.96)^*^	<0.001	<0.001
Predicted FVC (%)	84.57 (84.35–84.80)	92.04 (91.90–92.18)^*^	93.09 (92.93–93.23)^*^	<0.001	<0.001
FEV1/FVC	87.24 (87.11–87.37)	84.42 (84.33–84.50)^*^	83.26 (83.17–83.34)^*^	<0.001	<0.001
PEF (L/sec)	7.31 (7.28–7.34)	7.68 (7.66–7.69)^*^	7.88 (7.86–7.89)^*^	<0.001	<0.001

Data are presented as mean ± standard deviation.

*BMI*, body mass index; *FEV1*, Forced expiratory volume in first second; *FVC*, forced vital capacity; *PEF*, peak expiratory flow.

The multivariable model was adjusted for age, sex, height, status of smoking, vigorous exercise (times/week), diabetes, and high-sensitivity C-reactive protein. BMI categories: normal (between 18.5 and 25 kg/m^2^), underweight (<18.5 kg/m^2^), and obese (≥25 kg/m^2^).

^*^P < 0.001 compared to underweight.

**Figure 1:**
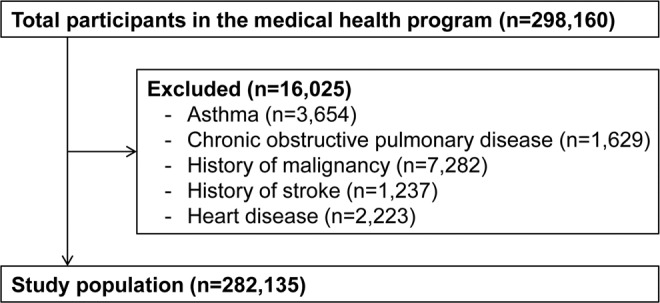
Study patient population.

**Figure 2:**
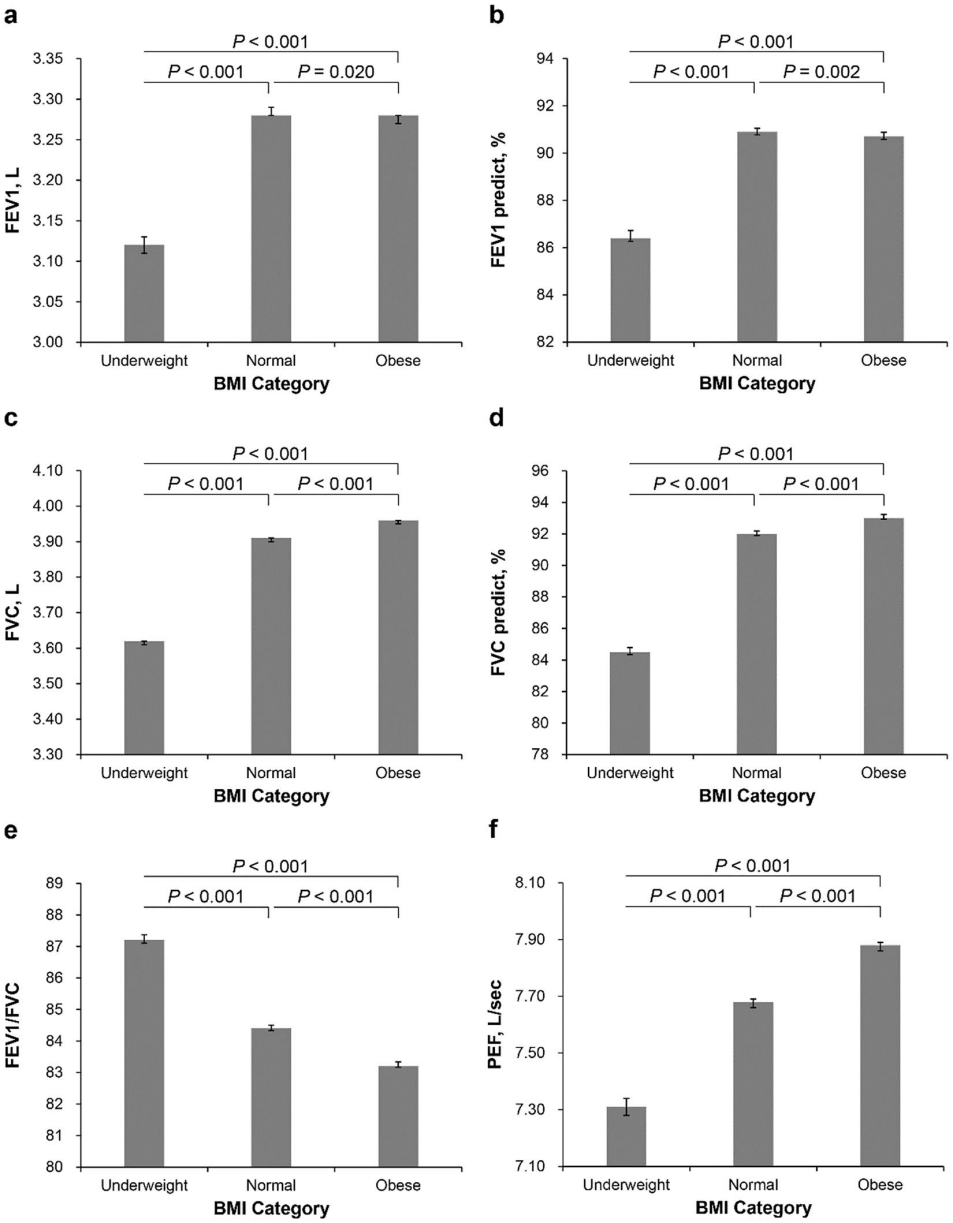
Adjusted mean values of pulmonary function parameters (95% CI) in the study group according to BMI. (**a**) FEV1 (L); (**b**) predicted FEV1 (%); (**c**) FVC (L); (**d**) predicted FVC (%); (**e**) FEV1/FVC; (**f**) PEF (L/sec). The multivariable model was adjusted for age, sex, height, status of smoking, vigorous exercise (times/week), diabetes, and high-sensitivity C-reactive protein. BMI categories: normal (between 18.5 and 25 kg/m^2^), underweight (<18.5 kg/m^2^), and obese (≥25 kg/m^2^).

**Figure 3:**
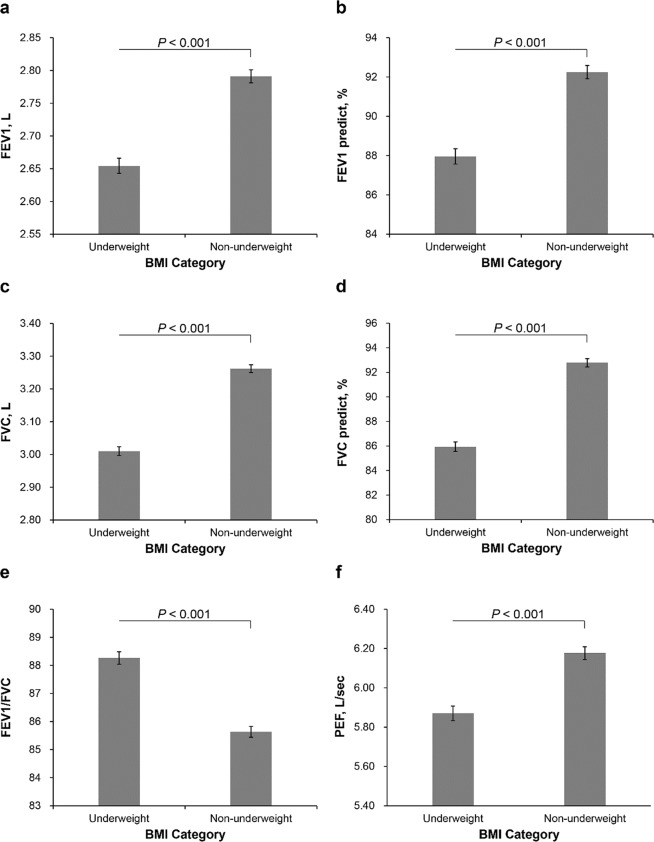
Adjusted mean values of pulmonary function parameters (95% CI) between underweight and non-underweight in women. (**a**) FEV1 (L); (**b**) predicted FEV1 (%); (**c**) FVC (L); (**d**) predicted FVC (%); (**e**) FEV1/FVC; (**f**) PEF (L/sec). The multivariable model was adjusted for age, height, status of smoking, vigorous exercise (times/week), diabetes, and high-sensitivity C-reactive protein. BMI categories: underweight (<18.5 kg/m^2^), non-underweight (≥18.5 kg/m^2^).

**Figure 4:**
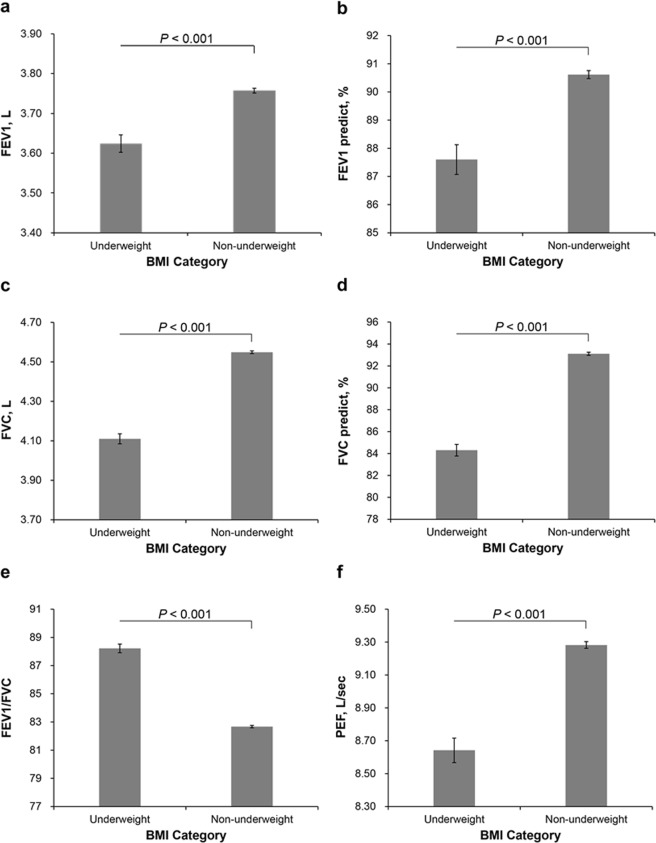
Adjusted mean values of pulmonary function parameters (95% CI) between underweight and non-underweight in men. (**a**) FEV1 (L); (**b**) predicted FEV1 (%); (**c**) FVC (L); (**d**) predicted FVC (%); (**e**) FEV1/FVC; (**f**) PEF (L/sec). The multivariable model was adjusted for age, height, status of smoking, vigorous exercise (times/week), diabetes, and high-sensitivity C-reactive protein. BMI categories: underweight (<18.5 kg/m^2^), non-underweight (≥18.5 kg/m^2^).

To clarify the effect of being underweight, subjects were divided into two groups: underweight (<18.5 kg/m^2^) and non-underweight (≥18.5 kg/m^2^). After adjusting for multiple covariates, the underweight group had lower FEV1 (L), predicted FEV1 (%), FVC (L), predicted FVC (%), and PEF (L/sec) but higher FEV1/FVC compared to the non-underweight group regardless of sex (Figs 3 and 4).

Table 4 presents results of univariate and multivariate logistic regression analysis between BMI and subjects with low pulmonary function parameters. After adjusting for possible confounding factors, adjusted ORs (95% CI) for subjects with predicted FEV1% < 80% for underweight and normal weight groups compared to obese group (reference) were 2.10 (1.98–2.21), and 0.93 (0.90–0.97), respectively (*P* for trend <0.001). Adjusted ORs for subjects with predicted FVC% < 80% for underweight and normal weight groups compared to obese group were 4.90 (4.62–5.18) and 1.32 (1.27–1.38), respectively (*P* for trend <0.001). Adjusted ORs for subjects with FEV1/FVC < 70% for underweight and normal weight groups compared to obese group were 0.95 (0.75–1.19) and 1.14 (1.06–1.23), respectively (*P* for trend <0.01).

**Table 4:** Crude and multivariate-adjusted ORs (95% CI) for decreased pulmonary function parameters according to BMI.

BMI category	Crude OR (95% CI)	Multivariate-adjusted OR (95% CI)
Predicted FEV1% < 80%		
Obese	1.00 (reference)	1.00 (reference)
Normal	0.872 (0.81–0.90)	0.93 (0.90–0.97)
Underweight	1.77 (1.69–1.85)	2.10 (1.98–2.21)
*P* for trend	<0.001	<0.001
Predicted FVC% < 80%		
Obese	1.00 (reference)	1.00 (reference)
Normal	1.58 (1.52–1.64)	1.32 (1.27–1.38)
Underweight	8.44 (8.04–8.86)	4.90 (4.62–5.18)
*P* for trend	<0.001	<0.001
FEV1/FVC < 70%		
Obese	1.00 (reference)	1.00 (reference)
Normal	0.86 (0.81–0.91)	1.14 (1.06–1.23)
Underweight	0.35 (0.29–0.41)	0.95 (0.75–1.19)
*P* for trend	<0.001	0.009

*BMI*, body mass index; *FEV1*, Forced expiratory volume in first second; *FVC*, forced vital capacity.

The multivariable model was adjusted for age, sex, height, status of smoking, vigorous exercise (times/week), diabetes, and high-sensitivity C-reactive protein. BMI categories: normal (between 18.5 and 25 kg/m^2^), underweight (<18.5 kg/m^2^), and obese (≥25 kg/m^2^).

## Discussion

In this cross-sectional study of the general population without apparent lung disease, we found that underweight was closely associated with decreased pulmonary function. The present study demonstrated that decreased pulmonary function represented by parameters such as FEV1, predicted FEV1 (%), FVC, predicted FVC (%), and PEF was associated with the underweight status even after adjusting for multiple covariates. Furthermore, the prevalence of subjects with predicted FEV1% < 80% and predicted FVC% < 80% were significantly associated with underweight even after adjusting for possible confounding factors. There is a proportional relationship between pulmonary function and the degree of BMI, consistent with previous studies^12–14^. Several studies have reported the relationship between underweight and pulmonary function in healthy subjects. Fukuhara *et al*. have found that predicted FEV1 is negatively correlated with BMI in 8,662 subjects aged over 40 years^13^. Another study on 370 university students has found that the underweight group has significantly lower FVC than the normal weight group^14^. A study on children and adolescents has also shown that underweight participants have lower predicted FVC (%) and vital capacity^12^. In a community-based study of 770 subjects, spirometry parameters such as FEV1, FVC, and PEF are significantly lower in underweight (BMI < 18.5 kg/m^2^) subjects than in normal weight (18.5 kg/m^2^ ≤ BMI ≤ 24 kg/m^2^) subjects^15^. The present study has several strengths, including robust number of participants, well defined underweight definition, and adjustments of multiple confounding factors that could affect pulmonary function.

Our present study found that underweight was associated with decreased pulmonary function parameters such as decreased FEV1, predicted FEV1 (%), FVC, predicted FVC (%), and PEF. There are several possible reasons why underweight status is related with decreased pulmonary function. Low muscle mass in underweight subjects is one of possible reasons^16^. Several studies have reported that low pulmonary function is related to reduced skeletal muscle mass^17,18^. Especially, diaphragmatic muscle mass has been reported to be reduced in an underweight population^19^. Theoretically, loss of intercostal and abdominal muscle mass could affect respiratory muscle strength and force. In a study of adult population, low pulmonary functions, FVC, and FEV1 are associated with low muscle mass^17^. That study showed that those with low FEV1 and FVC were more likely to have low muscle mass (OR = 3.11, 95% CI: 1.62–5.99 for FEV1; OR = 1.99, 95% CI: 1.13–3.53 for FVC) in men. From this point of view, low muscle mass of underweight population could result in low pulmonary function. Another possible reasons for low pulmonary function in the underweight population is physical inactivity. Physical activity can improve pulmonary function, maintain chest mobility, and counteract the loss of muscle mass. Vigorous physical activity can strengthen respiratory muscles. Moreover, deep breathing during exercise leads to full expansion of the diaphragm on inhalation and uses abdominalis muscles on exhalation, resulting in larger lung capacity. Physically inactive individuals are more likely to have low muscle mass and strength^20^. A study of sedentary young females has found that lean body mass is significantly correlated with low FVC and FEV1^11^. Reduced physical activity induces loss of protein synthesis, leading to loss of respiratory and diaphragm muscle. In our study, the frequency of vigorous exercise per week was lower in the underweight group than in other groups. In this regard, low muscle mass and physical inactivity might be associated with decreased pulmonary function in the underweight group.

The relationship between body weight and risk of mortality is an important concern in public health implication. Being underweight with an acute respiratory disease is associated with high mortality, especially for patients with ALI and ARDS. A recent meta-analysis study has pooled data from 6268 patients enrolled in five studies to determine the relationship between BMI and acute outcomes in patients with ARDS^8^. It found that being underweight was associated with higher mortality, while obesity and morbid obesity were more likely to result in lower mortality compared to those having normal weight^8^. Possible mechanisms of higher mortality risk in underweight patients with ALI/ARDS might be due to their deficits of metabolic reservation to counteract with increased catabolic stress under respiratory distress condition and their relatively poor basal respiratory function. On the other hand, it has been reported that obesity causes a decrease in mortality. This is referred to as the obesity paradox which exists in the respiratory system^21^. Hormonal changes, pro-inflammatory reaction, and immune-related activities such as adipose-triggered inflammatory mediators could peripherally alter physiological responses to injury and contribute to an acute respiratory reaction^22,23^. This present study revealed that pulmonary function parameters in obese subjects were higher than those in underweight subjects. Higher pulmonary function in obese subjects might prevent pulmonary deterioration when respiratory distress occurs. However, obese patients tended to have more medical morbidities and metabolic problems such as diabetes, hyperlipidemia, and hypertension. These comorbid illness and metabolic dysfunctions in obese population could affect their outcomes in acute illness state. Well designed, prospective studies are needed to elucidate the relationship between BMI and interaction of disease outcomes in clinical setting.

In this cross-sectional study, we assessed epidemiologic, clinical characteristics, and pulmonary function parameters simultaneously. There is a study that revealed longitudinal association of BMI with lung function^24^. Thyagarajan *et al*. has reported that, although the low BMI group has lower pulmonary function at baseline, the low BMI group has sustained lung function compared to other groups at 10 year follow up^24^. The lowest BMI quartile group (baseline BMI < 21.3 kg/m^2^) experienced 10 year increases of 71 ml in FVC and 60 ml in FEV1. In contrast, the highest BMI group (baseline BMI ≥ 26.4 kg/m^2^) experienced 10 year decreases of 185 ml in FVC and 64 ml in FEV1. However, the lowest BMI quartile of their study differs from BMI of underweight group in our study. In the lowest BMI quartile group (<21.3 kg/m^2^) of the study by Thyagarajan *et al*., 83.6% (990 of 1183) subjects had BMI ranging from 18.5 to 24.9 kg/m^2^. They considered these 83.6% (990 of 1183) subjects as the lowest BMI quartile group (<21.3 kg/m^2^). But, in our study, they were defined as normal BMI group. Different definition of the lowest BMI group might have led to different results and conclusion. Therefore, further cohort study is needed to investigate longitudinal changes of pulmonary function according to BMI.

Our study revealed that FEV1/FVC was notably decreased in obesity population. It is known that obese individuals have higher risk of respiratory impairments such as breathlessness and airway dysfunction. Several studies have investigated the association between obesity and pulmonary function^1,4,25^. Obesity may cause adverse effect on the respiratory system due to alteration in gas exchange, respiratory mechanism, and respiratory control^3^. Obesity affects respiratory function via several mechanisms, including mechanical factors of chest wall, abdomen, and upper airway. Obese people have deposition of fat tissue over the rib cage and abdomen, thus restricting chest wall movement^1,2^. The effect of obesity on FEV1/FVC is controversial. A multicenter cohort study on healthy young adults has found that FEV1/FVC is increased with increasing BMI^24^. However, Wang *et al*. have reported that FEV1/FVC is not affected by BMI^15^. One of possible reasons is that both FEV1 and FVC are affected at the same rate in obese subjects. Thus, the FEV1/FVC ratio can be preserved. However, in our study, FEV1/FVC was lower in the obese group than that in other groups. This finding is consistent with a previous study showing that obese individuals have lower FEV1/FVC values than younger age groups^26^. Davidson *et al*. have analyzed data from 327 healthy children and adolescents and reported that obese participants have lower FEV1/FVC than underweight participants^12^. Obese individuals might have smaller caliber airways due to fat tissue, resulting in reduced air way flow. Furthermore, abnormal production of cytokines and bioactive mediators in obese subjects can lead to a pro-inflammatory state and increase airway resistance^1,2^. These reasons could be associated with decreased airway function in obese population.

This study has several limitations. First, this was a cross-sectional study, not a longitudinal trial. There is predictive limitation in cross-sectional study design because epidemiologic, clinical characteristics, and pulmonary function parameters are simultaneously assessed. Prospective longitudinal follow-up study might be needed to investigate longitudinal changes of pulmonary function according to BMI. However, a robust sample size, well-designed and clustered structure of the sample are strengths of this study. Second, although the relationship between pulmonary function and BMI was evaluated, body composition components such as skeletal muscle mass or fat free mass was not considered. Lastly, we did not distinguish nutrition state in underweight population. Our study participants were generally healthy population, it could be difficult to find out mechanisms underlying the lung function-underweight association based on malnutrition from our study. Further study is needed to assess the effect of malnutrition on pulmonary function in underweight population. Despite these limitations, our study showed relationships between BMI and pulmonary function in a general population.

In conclusion, underweight status was found to be associated with decreased pulmonary function parameters such as FEV1 (L), predicted FEV1 (%), FVC (L), predicted FVC (%), and PEF (L/sec), but not with FEV1/FVC. Our results revealed that underweight status was independently associated with decreased pulmonary function in Korean population.

## Material and Methods

### Study design and patient population

This cross-sectional study was conducted to investigate the association between pulmonary function and underweight status represented by BMI. A total of 298,160 subjects (age range, 18–94 years old) who had undergone pulmonary function test in medical health check-up program held at Total Healthcare Center, Kangbuk Samsung Hospital, Seoul, South Korea between January 2012 and December 2014 were enrolled in this study. Of them, 16,025 subjects were excluded (3,654 subjects had asthma, 1,629 subjects had chronic obstructive pulmonary disease, 7,282 subjects had a history of malignancy, 1,237 subjects had a history of stroke, and 2,223 subjects had a history of coronary artery disease and/or congestive heart failure). The total number of eligible participants was 282,135 (Fig. 1).

### Anthropometric and laboratory measurements

We included the following variables in our analyses: medical history, information obtained from the self-administered questionnaire, anthropometric measurements, and laboratory measurements. Medical history and medicine prescription history were assessed by the examining physicians. All study participants were asked to respond to a smoking and exercise related questionnaire which included the status of smoking, the amount of smoking, and the frequency of vigorous exercise per week. Vigorous exercise was defined as an activity that required a large amount of effort and caused rapid breathing and a substantial increase in heart rate. Diabetes mellitus was defined as participant ever having been diagnosed with diabetes or current use of blood glucose lowering agents. Hypertension was defined as either participant ever having been diagnosed with hypertension or current use of antihypertensive medication. BMI was calculated by dividing weight (kg) by the square of height (m^2^). The study population was classified into three groups according to the degree of obesity using the Asian-Pacific obesity classification guideline. BMI categories were as follows: normal (between 18.5 kg/m^2^ and 25 kg/m^2^), underweight (<18.5 kg/m^2^), and obese (≥25 kg/m^2^).

Laboratory tests included fasting glucose, glycated hemoglobin, lipid profile, chemical profile, and hsCRP. Blood samples were collected from antecubital veins from participants who had fasted for at least 12 hours. Fasting serum glucose level was measured using the hexokinase method. HbA1c was measured using an immunoturbidimetric assay with a Cobas Integra 800 automatic analyzer (Roche Diagnostics, Basel, Switzerland). Total cholesterol and triglyceride levels were measured using enzymatic colorimetric tests. LDL-C was measured using the homogeneous enzymatic colorimetric test and high-density lipoprotein cholesterol (HDL-C) was measured using the selective inhibition method (Advia 1650 Auto-analyzer, Bayer Diagnostics; Leverkusen, Germany). Fasting insulin concentration was measured with immuno-radiometric assay (Biosource, Nivelles, Belgium).

### Pulmonary function measurements

Pulmonary function was measured by using a spirometer according to the criteria of the American Thoracic Society and the European Respiratory Society for standardization using a Vmax22 system (Sensor-Medics, Yorba Linda, CA, USA). FEV1 (L), FVC (L), predicted FEV1 (%), predicted FVC (%), PEF (L/sec), and FEV1/FVC were considered as pulmonary function parameters. Predicted FEV1 (%) and predicted FVC (%) were referred to previously established methods derived from the Korean population^27^. The following cut-off levels were used to determine low pulmonary function: FEV1 (%) < 80% of the predicted value, FVC (%) < 80% of the predicted value, and FEV1/FVC < 70%^18,28^. Information was examined carefully and compared with criteria metrics for acceptability, reproducibility, and quality control.

### Statistical analyses

Data are presented as mean ± standard deviation within BMI groups for continuous variables and as proportions for categorical variables. Main clinical characteristics and pulmonary function parameters among BMI groups were compared using ANOVA for continuous variables and Chi-square test for categorical variables. To clarify the effect of being underweight, subjects were divided into two groups: underweight and non-underweight between sexes. In order to test mean differences between study groups after adjusting for covariates (age, sex, height, status of smoking, frequency of vigorous exercise, diabetes, and hsCRP), adjusted mean values and standard deviation of pulmonary function parameters were calculated using ANCOVA. Univariate and multivariate logistic analysis were conducted to determine the association between BMI and pulmonary function parameters. Odds ratios (ORs) were calculated to determine risk for subjects with low PFT parameters [predicted FEV1 (%) < 80%, predicted FVC (%) < 80%, and FEV1/FVC < 70%] in underweight and normal weight groups compared with obese group (reference). All ORs were calculated with 95% confidence intervals (CIs). SPSS version 24.0 (IBM Corp., Chicago, IL, USA) was used for all statistical analyses and a P value less than 0.05 was considered statistically significant.

Ethics approval for the study protocol and analysis of the data were obtained from the Institutional Review Board of Kangbuk Samsung Hospital. This study was conducted in accordance with the 1975 Declaration of Helsinki. The requirement of obtaining informed consent was waived by the Board because we accessed a de-identified database retrospectively for data analysis. There were no commercial conflicts of interest related to this study.

## Data Availability

Datasets used for the current study are not available publicly as this was not specified in the original ethical approval request. However, they may be available in part from the corresponding author upon reasonable request.
